# Age, sex and (the) race: gender and geriatrics in the ultra-endurance age

**DOI:** 10.1186/2046-7648-3-1

**Published:** 2014-01-01

**Authors:** Greg Whyte

**Affiliations:** 1 Research Institute for Sport & Exercise Science, Liverpool John Moores University, Liverpool L3 3AF, England

**Keywords:** Ultra-endurance, Extreme conditions, Ultra-marathon, Performance trends, Sport, Gender differences

## Abstract

Ultra-endurance challenges were once the stuff of legend isolated to the daring few who were driven to take on some of the greatest physical endurance challenges on the planet. With a growing fascination for major physical challenges during the nineteenth century, the end of the Victorian era witnessed probably the greatest ultra-endurance race of all time; Scott and Amundsen’s ill-fated race to the South Pole. Ultra-endurance races continued through the twentieth century; however, these events were isolated to the elite few. In the twenty-first century, mass participation ultra-endurance races have grown in popularity. Endurance races once believed to be at the limit of human durability, i.e. *marathon running*, are now viewed as middle-distance races with the accolade of true endurance going to those willing to travel significantly further in a single effort or over multiple days. The recent series of papers in *Extreme Physiology & Medicine* highlights the burgeoning research data from mass participation ultra-endurance events. In support of a true ‘mass participation’ ethos Knetchtle et al. reported age-related changes in Triple and Deca Iron-ultra-triathlon with an upper age of 69 years! Unlike their shorter siblings, the ultra-endurance races appear to present larger gender differences in the region of 20% to 30% across distance and modality. It would appear that these gender differences remain for multi-day events including the ‘Marathon des Sables’; however, this gap may be narrower in some events, particularly those that require less load bearing (i.e. swimming and cycling), as evidenced from the ‘Ultraman Hawaii’ and ‘Swiss Cycling Marathon’, and shorter (a term I used advisedly!) distances including the Ironman Triathlon where differences are similar to those of sprint and endurance distances i.e. *c.* 10%. The theme running through this series of papers is a continual rise in participation to the point where major events now require selection races to remain within reasonable limits. With the combination of distance and environment placing a significant physiological bordering on pathophysiological burden on the participants of such events, one question remains: Are we destined for another Scott vs*.* Amundsen? How long is too long?

## Editorial

Ultra-endurance challenges were once the stuff of legend isolated to the daring few who were driven to take on some of the greatest physical endurance challenges on the planet. It was the Victorians, however, that accelerated the quest to pit man against nature transforming the primary purpose of these challenges from discovery to racing. The end of the Victorian era witnessed probably the greatest ultra-endurance race of all time; Scott and Amundsen’s ill-fated race to the South Pole. Ultra-endurance races continued through the twentieth century; however, these events were isolated to the elite few. Of note, arguably the greatest ultra-endurance race of the modern era, the Tour de France, has been in existence since 1903 and celebrates its 100th anniversary this year.

Today, ultra-endurance events have become commonplace, almost routine, with scores of people taking on some of the most iconic challenges on the planet i.e. Everest, North and South Pole and swimming the English Channel. In contrast, mass participation ultra-endurance races are a relatively recent phenomenon emerging over the last quarter of a century. Endurance races once believed to be at the limit of human durability, such as marathon running, are now viewed as middle-distance races with the accolade of true endurance going to those willing to travel significantly further in a single effort or over multiple days. Unlike their older, but shorter, endurance siblings, modern ultra-endurance races have developed without gender bias, offering equality since their inception. It is always valuable to remind ourselves that women were banned from racing in running events longer than 800 m until the mid-1960s and the first Olympic Marathon for women was not held until the Los Angeles Olympics of 1984! The primary reason for these thresholds were the beliefs of the misogynous ‘blazer brigade’ of the sport’s governing bodies that women would irreparably damage their reproductive system if they were to compete in such prolonged physical exertion. Sadly, it was the non-evidenced-based guidance of medics and scientists of the day that provided support for this gender apartheid, something that the Sport Science and Medicine research of today has unequivocally refuted. Indeed, recently, a number of researchers have suggested that women will outperform men in the near future. These suggestions have been made based on weak analysis using linear regression modelling—a method that suggests both men and women will travel self-propelled and faster than the speed of light at some time in the future! Recent work from our group [1,2] and others [3] has demonstrated that women are, and will likely remain, slower than their male counterparts with a strangely consistent 10% difference in speed across distance and modality (run, swim, cycle, triathlon) for sprint and endurance race distances.

A recent series of papers in *Extreme Physiology & Medicine* highlights the burgeoning research data from ultra-endurance and provides a collection of studies examining the breadth of mass participation from the single-stage Deca Iron ultra-triathlon (3.8-km swim, 1,800-km cycle, 420-km run!) to the multi-day running races of the Marathon de Sables. In support of a true ‘mass participation’ ethos Knetchtle et al. [4] reported age-related changes in Triple and Deca Iron-ultra-triathlon with an upper age of 69 years! Of note, the authors demonstrated the winners of these events were in their second to fourth decade of life (25 to 44 years). In line with all available physiological data, performance declined with age thereafter. Zingg et al. [5] supported this finding in the 24-h ultra-marathon runners with a mean age for the peak running speed in the late third to early fourth decade of life in both male and female competitors—a finding supported in Ironman-triathletes who peak at 32–33 years of age [6]. The reason for the older age of elite performers is not clear but is likely associated with a range of factors underpinned by experience, in particular, pace judgement and nutritional and race strategies.

Unlike their shorter siblings, the ultra-endurance races appear to present larger gender differences in the region of 20% to 30% across distance and modality. Sigg et al. [7] reported a 23.7% ± 13.1% difference between men and women over the double Iron ultra-triathlon, a figure supported from other papers in this series over different distances and modalities [4,5]. Whilst these gender differences appear to remain for multi-day events including the ‘Marathon des Sables’ [8], they may be narrower in some events—particularly those that require less load bearing (e.g. swimming and cycling). Thus, gender differences in the ‘Ultraman Hawaii’ [9], ‘Swiss Cycling Marathon’ [10], and the shorter (a term I used advisedly!) Ironman Triathlon [11] are similar to those of sprint and endurance distances (*c.* 10%). Whilst the origin of such gender differences is multi-factorial, there are two key physiological factors that differentiate men from women: VO_2max_ and power production. Women have a lower oxygen carrying capacity (*c.* 10% lower haemoglobin), smaller hearts (lower Q) and lungs (lower diffusion capacity) even when scaled for body size, and a higher body fat percentage resulting in a lower aerobic power. Furthermore, women have lower peak strength and power resulting in a lower peak velocity [12].

Whilst gender differences in performance remain, the same cannot be said for participation. Unhindered by Victorian middle class, male-dominated governing bodies of sport, the new ultra-endurance era has witnessed an exponential growth in participation with gender equality. The theme running through this series of papers is a continual rise in participation to the point where major events now require selection races to remain within reasonable limits of scale. With the combination of distance and environment [13] placing a significant physiological (bordering on pathophysiological) burden on the participants of such events, one question remains: Are we destined for another Scott vs*.* Amundsen? How long is too long?

## Competing interests

The author declares that he has no competing interest.
